# Intrinsically Disordered Flanking Regions Increase the Affinity of a Transcriptional Coactivator Interaction across Vertebrates

**DOI:** 10.1021/acs.biochem.3c00285

**Published:** 2023-08-30

**Authors:** Elin Karlsson, Carl Ottoson, Weihua Ye, Eva Andersson, Per Jemth

**Affiliations:** Department of Medical Biochemistry and Microbiology, Uppsala University, BMC Box 582, SE-75123 Uppsala, Sweden

## Abstract

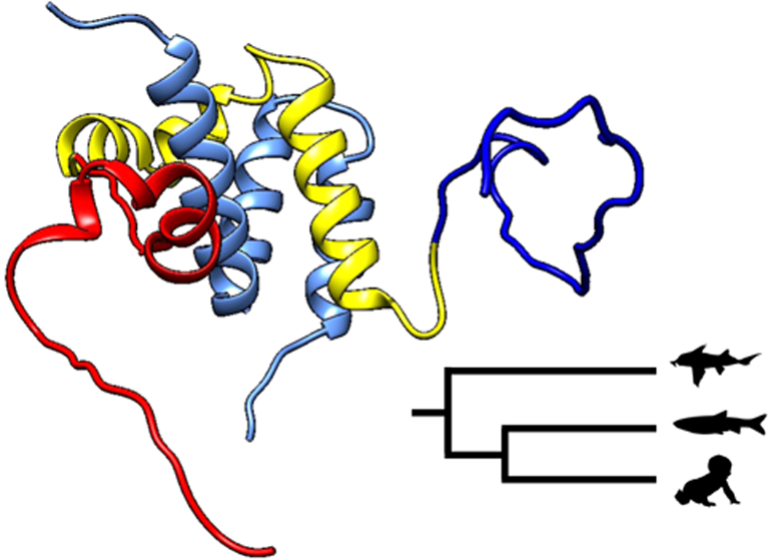

Interactions between two proteins are often mediated by a disordered region in one protein binding to a groove in a folded interaction domain in the other one. While the main determinants of a certain interaction are typically found within a well-defined binding interface involving the groove, recent studies show that nonspecific contacts by flanking regions may increase the affinity. One example is the coupled binding and folding underlying the interaction between the two transcriptional coactivators NCOA3 (ACTR) and CBP, where the flanking regions of an intrinsically disordered region in human NCOA3 increases the affinity for CBP. However, it is not clear whether this flanking region-mediated effect is a peculiarity of this single protein interaction or if it is of functional relevance in a broader context. To further assess the role of flanking regions in the interaction between NCOA3 and CBP, we analyzed the interaction across orthologs and paralogs (NCOA1, 2, and 3) in human, zebra fish, and ghost shark. We found that flanking regions increased the affinity 2- to 9-fold in the six interactions tested. Conservation of the amino acid sequence is a strong indicator of function. Analogously, the observed conservation of increased affinity provided by flanking regions, accompanied by moderate sequence conservation, suggests that flanking regions may be under selection to promote the affinity between NCOA transcriptional coregulators and CBP.

## Introduction

**Figure 1:**
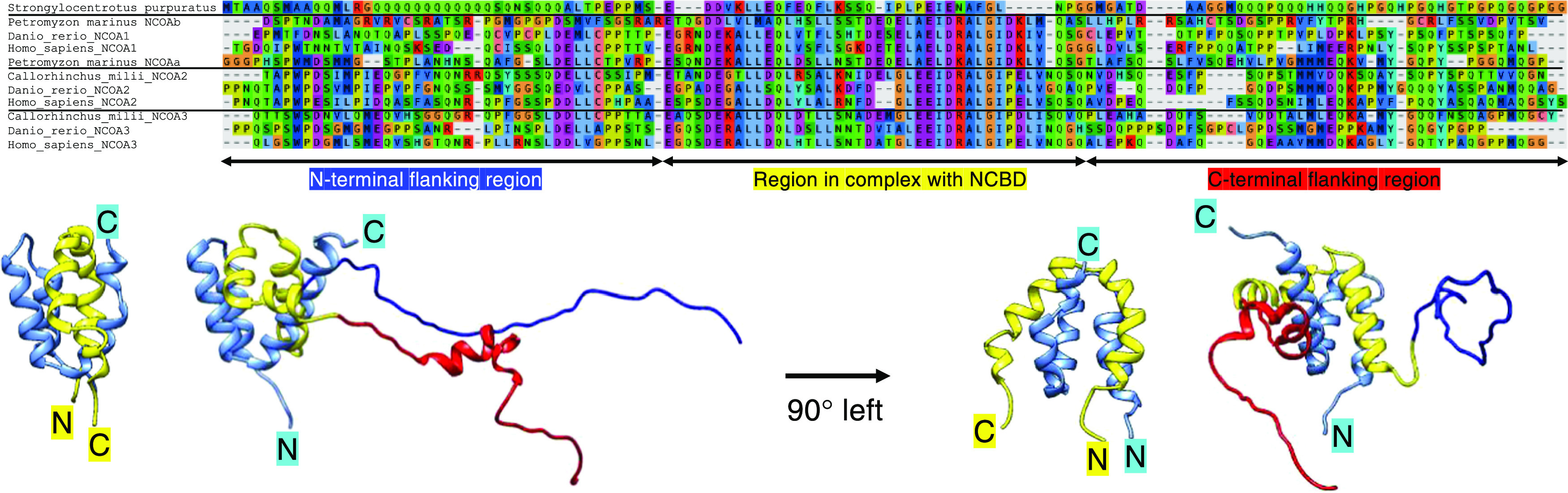
Sequence alignment of CID domains with flanking regions and structural models of the NCBD/CID complex. The sequence identity within the CID region that forms the interface with NCBD is higher than that in the flanking regions. The complex between human NCBD and NCOA3 CID with flanking regions (N, dark blue; C, red) (residues 1006–1125) was predicted by ColabFold.^16^ The predicted complex is compared with a complex solved by NMR (Protein Data Bank ID 6ES7)^17^ with the short NCOA3 CID (residues 1045–1084) and a slightly shorter NCBD construct than that used in the present paper, corresponding to the conserved region, which binds the CID domain (residues 2062–2109). There are differences in the conserved regions, most notably in the C-terminal helix of NCOA3 CID. This may be due to both uncertainty in the prediction (IDDT ∼70–80 in the helical regions) and an inherent flexibility in NCBD/CID complexes.^17^ In either case, the N- and C-terminal flanking regions are predicted as intrinsically disordered (IDDT ∼40).

The major determinants for a specific protein–protein interaction are found in the binding interface between the two proteins, as shown in numerous structural studies in combination with mutagenesis and binding assays. Presently, the field of systems biology grapples with how to integrate data on individual interactions in the context of the living cell. In addition to all specific interactions, *i.e*., those under natural selection for fitness, it is clear that nonspecific interactions between proteins and quinary interactions^1,2^ affect the stability and function of proteins. Moreover, apparently nonspecific interactions within a protein complex add another layer of complexity. Such interactions usually involve intrinsically disordered regions outside of the ordered binding interface, and they may form short-lived nonspecific interactions with the surface of the ligand molecule modulating the affinity of the complex.^3−8^ We have recently investigated the role of flanking regions in the interaction between two transcriptional coactivators, CREB-binding protein (CBP), and nuclear receptor coactivator 3 (NCOA3, also called ACTR).^9^ The interaction domains are the molten globule-like nuclear coactivator binding domain (NCBD)^10−13^ of CBP and the highly disordered CBP-interacting domain (CID) of NCOA3, which interact in a coupled binding and folding reaction, resulting in a well-ordered complex.^14,15^ The flanking disordered regions of the human CID domain from NCOA3 increases the affinity 3-fold for NCBD, likely via nonspecific hydrophobic or polar interactions.^9^ This may seem a minor contribution in terms of free energy of binding (<1 kcal mol^–1^), but the effect can be substantial if present in larger protein complexes such that the combined effect of several interactions provides an overall significant affinity. The regions involved in forming the complex interface are well conserved, for both NCBD and CID, but more sequence changes have occurred outside of the binding regions (Figure 1).

While a high substitution rate is expected for intrinsically disordered linker regions without a dedicated function,^18^ nonconservation would question whether the influence of flanking regions on binding affinity is of any functional significance. To address this question, we measured the effect of flanking regions on the affinity for several NCBD/CID complexes. We selected CIDs from orthologs in different species but also from the two other paralogs present in vertebrates, NCOA1 (Src1) and NCOA2 (Tif1), respectively. Our data show that the affinity in complexes from three jawed vertebrate species separated by 420–450 million years of evolution is consistently increased by the intrinsically disordered regions that flank the CID region defined by the complex. Our data corroborate the notion of interacting flanking regions as a general way to modulate affinity in protein interactions, despite less stringent constraints on the amino acid sequence compared to the binding interface.

## Results and Discussion

### Phylogenetic Analysis of Flanking Regions around the CID Domain

We could previously identify the CID domain only in deuterostome animals (vertebrates, echinoderms, hemichordates) but not in protostomes (arthropods, nematodes, annelids, molluscs).^19^ Thus, we concluded that the CID domain and its interaction with CBP emerged in an ancestral deuterostome. In the present work, we therefore collected NCOA sequences from different deuterostome animals in the Uniprot and NCBI databases. Based on our study on flanking regions for CID in human NCOA3,^9^ we investigated 39 amino acid residues on either side of the “core” CID domain (Figure 1). The sequence alignment shows, as expected, that the flanking regions are less conserved than the core CID domain, which is defined by the binding interface with NCBD in NMR structures of the complex.^14,15,17^ Conservation of disordered flanking regions is not straightforward to define quantitatively in terms of identity because of multiple insertions and deletions. However, there are conserved features in the flanking regions, for example, a D–D/E–Φ–Φ motif at the end of the N-terminal flanking region. (This motif could serve as a binding partner for another, unidentified protein domain.) Furthermore, both the N- and C-terminal flanking regions from jawed vertebrates have calculated isoelectric points between 3.39 and 4.66 due to more Asp and Glu as compared to Lys and Arg residues (Supporting Information Text File 1). These conserved features suggest that the flanking regions may play a role beyond acting as linkers between functional domains.

Two whole genome duplications occurred in an early vertebrate around 450 million years ago,^20^ resulting in paralogs of many genes that are conserved in all present-day jawed vertebrates (gnathostomes). While the relationship between the three NCOA paralogs from jawed vertebrates is clear, the phylogeny of the nonjawed vertebrate *Petromyzon marinus* is not. All jawed vertebrates contain three paralogs, NCOA1, NCOA2, and NCOA3. Sequence-based phylogeny supports a scenario where the gene encoding NCOA1 diverged from the ancestral NCOA2/3 gene in the first whole genome duplication and NCOA2 and NCOA3 diverged in the second genome duplication. However, for *P. marinus*, when taking the full-length NCOA sequences into account, the two paralogs, here denoted NCOAa and NCOAb, do not clearly group with specific NCOA paralogs from the jawed vertebrates (Figure S1). It is not clear whether the nonjawed vertebrates diverged before, during, or after the two whole genome duplications in the jawed vertebrate lineages.^20,21^ Thus, NCOAa and NCOAb may have originated in the first genome duplication and experienced extensive sequence divergence-relative NCOA1 and NCOA2/3 or be the result of a separate gene duplication occurring after the split between jawed and nonjawed vertebrates.

### Experimental Interaction Studies between NCBD and CID

For binding experiments, we designed expression constructs for CID and NCBD domains from five animals, based on phylogeny and previous experiments:^19,22^*Strongylocentrotus purpuratus* (purple sea urchin, an echinoderm, see note in the Materials section), *P. marinus* (sea lamprey, a jawless vertebrate), *Callorhinchus milii* (Austrailan ghost shark, a cartilaginous fish), *Danio rerio* (zebra fish, a bony fish), and *Homo sapiens*, representing tetrapods (Figures 1 and S1). We have previously expressed and purified the short version of CID from these animals and from the human paralogs,^19,22^ but obtaining CID with flanking regions proved very challenging. Thus, while we initially aimed for four different expression constructs from each NCBD/CID complex consistent with our previous study^9^ (the longest N-CID-C with both flanking regions, the N-terminal flanking region N-CID, the C-terminal CID-C, and the minimal region CID), we had to resort to comparing only the longest N-CID-C with CID.

**Figure 2:**
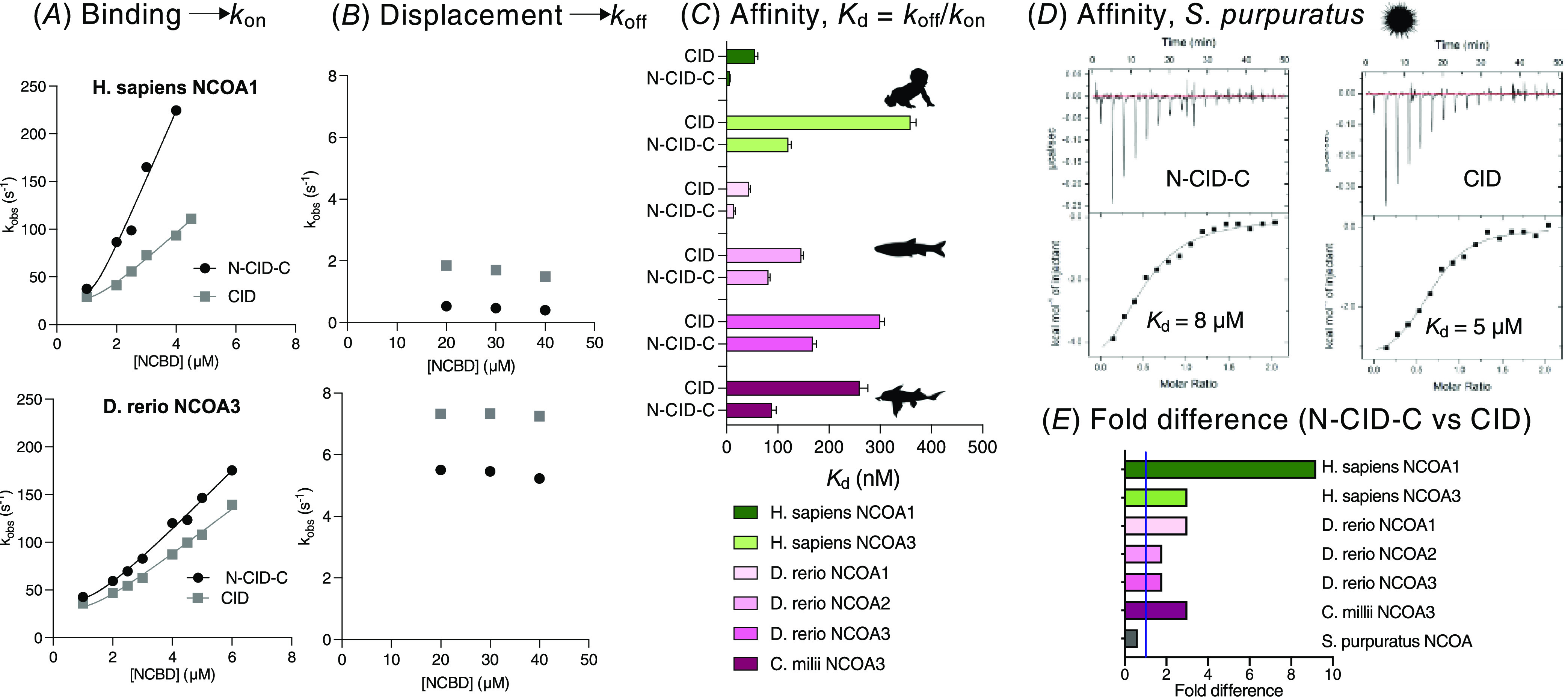
Determination of affinity using stopped-flow spectroscopy and isothermal titration calorimetry. (A) Examples of observed rate constants (*k*_obs_) from binding experiments between NCBD and CID from *H. sapiens* and *D. rerio*. *k*_obs_ values were plotted versus CID concentration, and the slope at high [NCBD] corresponds to the association rate constant *k*_on_. (B) The dissociation rate constant was measured in a separate displacement experiment where the dissociation of the NCBD/CID complex was induced by an excess of wild-type NCBD domain. The observed rate constant is a good approximation of the dissociation rate constant, and *k*_off_ was calculated as the average of the three experiments shown. The equilibrium constant *K*_d_ was calculated as the ratio of *k*_off_ and *k*_on_. Kinetic data from all experiments are shown in Figure S2 and the Supporting Information Excel File. (D) Isothermal titration calorimetry was used to determine *K*_d_ for the low-affinity interaction between NCBD and CID from *S. purpuratus*. (E) The difference in affinity between long (N-CID-C) and short (CID) variants is shown as fold difference. The blue vertical line is at fold difference = 1, i.e., corresponding to identical affinity.

We were able to express a long version (N-CID-C) and the minimal region (CID) from seven NCOAs: *H. sapiens* NCOA1, *H. sapiens* NCOA3, *D. rerio* NCOA1, *D. rerio* NCOA2, *D. rerio* NCOA3, *C. milii* NCOA3, and *S. purpuratus* NCOA. Except for *S. purpuratus* NCBD/CID, affinities were determined with stopped-flow spectroscopy using a Trp variant of NCBD from the respective species, as previously described^9^ (Figures 2 and S2).

**Figure 3:**
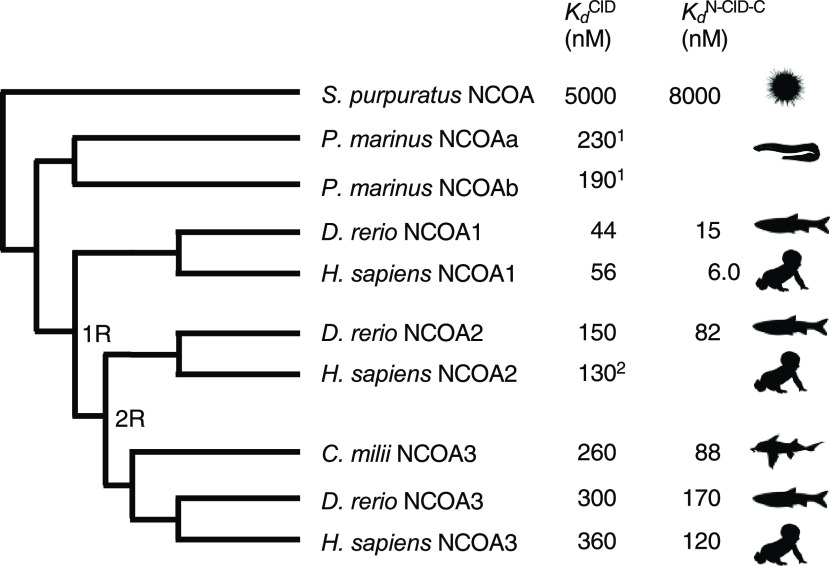
Affinities mapped on a phylogenetic tree. A simplified phylogeny with affinities of NCBD/CID complexes from the present and previous work (indicated by footnotes: ^1^Karlsson et al.^22^ and ^2^Hultqvist et al.^19^). *K*_d_ values derived from kinetic experiments have high precision, and the propagated errors from *k*_on_ and *k*_off_ are usually low (below 10%, see the Supporting Information Excel File). The fold difference between *K*_d_^CID^ and *K*_d_^N-CID-C^ for any particular pair is very accurate since the same NCBD solutions were used in stopped-flow experiments run back-to-back.

The independent determination of *k*_on_ and *k*_off_ by stopped-flow spectroscopy gives both high accuracy and precision to the data, which is important when comparing relatively small differences in the *K*_d_ value. For all of the vertebrate complexes, the affinity was increased by the presence of flanking regions, usually by 2–3-fold. However, for *H. sapiens* NCOA1 CID, the presence of flanking regions increased the affinity as much as 9-fold (Figures 2 and 3 and the Supporting Information Excel File). For the low-affinity nonvertebrate *S. purpuratus* NCBD/CID complex, we used isothermal titration calorimetry (ITC) to estimate the affinity. In this case, we did not observe a change in affinity from the flanking regions within the error of the ITC experiment, *K*_d_ = 8 and 5 μM with and without flanking regions, respectively (Figure 2). Furthermore, as we showed in a previous study, the stoichiometry of the *S. purpuratus* NCBD/CID interaction appears to be NCBD:CID 1:2, which complicates the analysis.^22^ We note that the sequence composition of the flanking regions in *S. purpuratus* CID is very different from that of the chordates, with multiple Gln residues and only one Glu in the N-terminal regions and one Asp in the C-terminal regions, resulting in higher calculated isoelectric points (Supporting Information Text File 1).

### Flanking Regions Remain Intrinsically Disordered in the Complex

**Figure 4:**
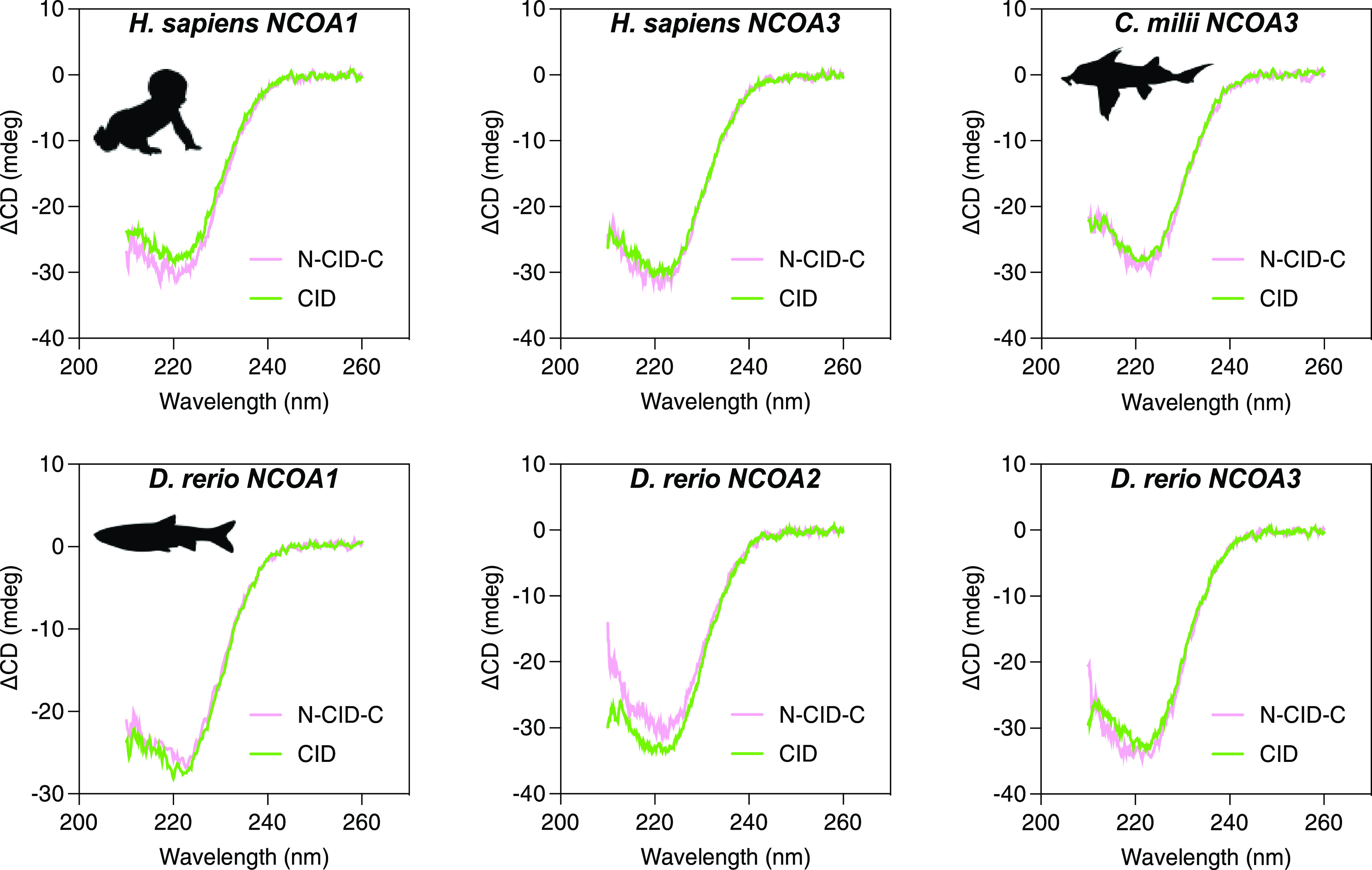
Difference spectra for CID/NCBD complexes. Difference spectra for vertebrate NCBD/CID complexes. Difference spectra are shown for both the long N-CID-C and the short CID constructs. The similar difference spectra between N-CID-C and CID suggest that the flanking regions do not fold into a particular secondary structure upon binding to NCBD.

The regions of CID that are in direct contact with NCBD in the complex fold to α helices upon binding (Figure 1).^15,17^ We performed circular dichroism (CD) experiments to estimate formation of helix upon binding, for the core CID region and for N-CID-C, for four complexes (Figure 4). Difference spectra between bound and free CID (or N-CID-C) show the increase in the CD signal associated with binding. Furthermore, the very similar changes for CID and N-CID-C suggest that it is only the core CID region that folds into helices and that the flanking regions remain intrinsically disordered. These results are consistent with previous data for human NCOA3 CID and NCBD across a range of ionic strength^9^ and with ColabFold prediction (Figure S3).

## Conclusions

It has become clear that protein interaction and stability depend on context including regions outside of the “canonical” binding site.^4,5,9,23−27^ Emerging quantitative data suggest a role of disordered flanking regions, which could make multiple transient interactions with a folded interaction partner to either increase or decrease affinity.^3,4,9^ Phylogenetic methods are powerful in pinpointing evolutionarily conserved regions in proteins. If these regions are involved in a protein–protein interaction, then the conserved residues are likely important for affinity and specificity. Intrinsically disordered regions in proteins are usually less conserved than ordered ones,^18^ although CID is an example of a very conserved disordered region. Obviously, the reason is that CID is directly involved in a binding interface with the NCBD domain of CBP and p300 and is therefore under strong selection to maintain the affinity of the complex. While the N- and C-terminal flanking regions of CID are less conserved than the “core” CID region in terms of sequence identity (Figure 1), we here show that they contribute to increasing the binding affinity in three different jawed vertebrate species (a shark, a bony fish, and a mammal), which diverged between 420 and 450 million years ago. But how is this apparently evolutionary conserved trait achieved? It is conceivable that the nonspecific interactions contributed by the flanking regions are less dependent on a specific amino acid sequence compared to those in the binding interface and more on sequence composition. For example, in the present case, there is a conserved motif including negatively charged residues in the N-terminal flanking region and additional relatively well conserved negative net charges in both the N- and C-terminal regions, as well as a Trp residue in the N-terminal. Our study on human NCOA3/NCBD demonstrated a lack of ionic strength dependence suggesting that charge–charge interactions are not involved in increasing the affinity.^9^ Thus, extrapolating to our present data, the flanking regions may be under purifying selection to maintain a certain degree of favorable polar or nonpolar nonspecific interactions, where the structural flexibility allows many combinations of residues. This is conceptually similar to the “conformational buffering” proposed based on experiments with adenovirus E1A and host Rb protein, where overall properties rather than exact sequence are conserved.^28^ It could be argued that the observed effects on affinity are coincidental and of no functional importance. Because of the huge sequence space of even short disordered flanking regions, this objection is hard to refute since there will always be sequences that either promote or reduce affinity in a given context. In other words, negative controls are hard to design, and experiments would be difficult to interpret. While our approach, investigating the effect of naturally evolved and related sequences, does not provide direct proof, it corroborates the hypothesis that flanking regions promote interactions. In conclusion, our present data, limited to three paralogs and three species, and with the caveats delineated above, suggest that flanking regions are under selection for increasing the affinity and may therefore contribute functionally to the interaction between the transcriptional coregulator families CBP/p300 and NCOA in jawed vertebrates.

## Materials and Methods

### Bioinformatics

Protein sequences were downloaded from Uniprot or NCBI (Supporting Information Text Files 2 and 3). Sequence alignment was performed with ClustalO^29^ and Muscle.^30^ Overall, the regions corresponding to NCBD and CID are well conserved among animals.^19^ Sequences representing different branches of the deuterostome animal tree were selected for experiments. Prediction of complex structures were done by ColabFold,^16^ which builds on AlphaFold2.^31^ The sequence for *S. purpuratus* NCOA (W4YZZ7) was withdrawn from Uniprot at a late stage of the project and is now presented in UniParc (UPI000222AEB3). It is also present in NCBI and annotated as neurogenic protein mastermind (XP_030830181.1). A homologous sequence from the related *L. variegatus* (green sea urchin) is present in NCBI and annotated as NCOA2-like. Based on comparisons between these and the vertebrate NCOAs (Supporting Information Text File 3), we decided to keep the data for *S. purpuratus* in this paper.

### Expression and Purification

Expression constructs were ordered from Genscript. Each plasmid encoded a 6 His-tagged lipo domain, followed by a thrombin cleavage site and the protein of interest (a CID or NCBD variant). The Uniprot ID for each sequence is shown in Supporting Information Text File 2. Gly-Ser remains at the N-terminus after thrombin cleavage. N-CID-C and CID from *D. rerio* NCOA2 were expressed with a PreScission site to improve yield. Here, Gly-Pro-Gly-Ser remains after cleavage. The first four residues in NCBD from *C. milii* were truncated during expression and purification, as shown by Maldi-TOF mass spectrometry, and this truncated NCBD was used in the experiments. For kinetic studies, a Trp was introduced at the position corresponding to Tyr2108 in human NCBD.^32^ The expressed sequences are compiled in Supporting Information Text File 2. Expression and purification of CID and NCBD variants have been previously described in detail.^9^

### Biophysical Experiments

All experiments to assess the secondary structure and determine affinity were performed in 20 mM sodium phosphate (pH, 7.40, 150 mM NaCl). Far-UV circular dichroism spectra were recorded in a Jasco J-1500 spectropolarimeter with a 1 mm quartz cuvette (Figure 4). Kinetic experiments were performed in an instrument from applied photophysics at a low temperature (4 °C) to facilitate kinetic experiments by reducing the observed rate constants. The details of the kinetic experiments and analysis of data were recently published.^9^ To obtain the observed rate constant *k*_obs_ (Figures 2 and S2), kinetic transients were fitted to either a single exponential function or, in the case of human and *C. milii* NCOA3, a double exponential to account for a slow kinetic phase in displacement experiments likely associated with equilibration of two alternative complexes following initial binding.^33^ The high *k*_obs_ values were used to calculate *K*_d_. Isothermal titration calorimetry (ITC) experiments (Figure 2) were performed at 25 °C in a MicroCal iTC200 system (Malvern) as described in figure legends and in Karlsson et al.^22^

## Supporting Information Available

Phylogenetic gene tree and cladogram for the NCOA sequences; plots of all observed rate constants for CID/NCBD interactions; and confidence of colabfold prediction of the NCOA3 N-CID-C/NCBD complex (PDF)
Rate constants and calculations (XLSX)
pI values of N- and C-terminal flanking regions (TXT)
Protein sequences and calculated isoelectric points (TXT)
Full-length sequences (TXT)

## Accession Codes

S_purpuratus_NCBD_WT_2316-2374 Uniprot A0A7M7HNR9; P_marinus_NCBD_WT_841-899 Uniprot S4RJD3; C_milli_NCBD_WT_2031-2089 Uniprot A0A4W3HN69; D_rerio_NCBD_WT_2064-2122 Uniprot A0A8M9PNF9; H_sapiens_NCBD_WT_2058-2116 Uniprot Q92793; S_purpuratus_NCOA_N-CID-C_1037-1156 UniParc UPI000222AEB3 (previously Uniprot W4YZZ7); S_purpuratus_NCOA_CID_1076-1117 UniParc UPI000222AEB3 (previously W4YZZ7); P_marinus_NCOAa_N-CID-C_966-1085 Uniprot S4RC77; P_marinus_NCOAa_CID_1005-1046 Uniprot S4RC77; P_marinus_NCOAb_N-CID-C_938-1057 Uniprot S4RR69; P_marinus_NCOAb_CID_977-1018 Uniprot S4RR69; C_milli_NCOA2_N-CID-C_1067-1186 NCBI XP_007885122.1; C_milli_NCOA2_CID_1106-1147 NCBI XP_007885122.1; C_milli_NCOA3_N-CID-C_1037-1156 Uniprot A0A4W3K0P0; C_milli_NCOA3_CID_1076-1117 Uniprot A0A4W3K0P0; D_rerio_NCOA1_N-CID-C_830-949 Uniprot A0A8M9PTD7; D_rerio_NCOA1_N-CID-C_869-910 Uniprot A0A8M9PTD7; D_rerio_NCOA2_N-CID-C_1020-1139 Uniprot Q98TW1; D_rerio_NCOA2_CID_1059-1100 Uniprot Q98TW1; D_rerio_NCOA3_N-CID-C_1055-1174 Uniprot F1QMV7; D_rerio_NCOA3_CID_1094-1135 Uniprot F1QMV7; H_sapiens_NCOA1_N-CID-C_885-1004 Uniprot Q15788; H_sapiens_NCOA1_CID_924-965 Uniprot Q15788; H_sapiens_NCOA2_N-CID-C_1030-1149 Uniprot Q15596; H_sapiens_NCOA2_CID_1069-1110 Uniprot Q15596; H_sapiens_NCOA3_N-CID-C_1006-1125 Uniprot Q9Y6Q9; H_sapiens_NCOA3_CID_1045-1086 Uniprot Q9Y6Q9.

## References

[ref1] Cohen R. D.; Pielak G. J. A cell is more than the sum of its (dilute) parts: A brief history of quinary structure. Protein Sci. 2017, 26, 403–413. 10.1002/pro.3092. 27977883 PMC5326556

[ref2] Vallina Estrada E.; Oliveberg M. Physicochemical classification of organisms. Proc. Natl. Acad. Sci. U.S.A. 2022, 119, e2122957119 10.1073/pnas.2122957119. 35500111 PMC9171632

[ref3] Troilo F.; Bonetti D.; Bignon C.; Longhi S.; Gianni S. Understanding Intramolecular Crosstalk in an Intrinsically Disordered Protein. ACS Chem. Biol. 2019, 14, 337–341. 10.1021/acschembio.8b01055. 30715849

[ref4] Staby L.; Due A. D.; Kunze M. B. A.; Jørgensen M. L. M.; Skriver K.; Kragelund B. B. Flanking Disorder of the Folded αα-Hub Domain from Radical Induced Cell Death1 Affects Transcription Factor Binding by Ensemble Redistribution. J. Mol. Biol. 2021, 433, 167320 10.1016/j.jmb.2021.167320. 34687712

[ref5] Theisen F. F.; Staby L.; Tidemand F. G.; O’Shea C.; Prestel A.; Willemoës M.; Kragelund B. B.; Skriver K. Quantification of Conformational Entropy Unravels Effect of Disordered Flanking Region in Coupled Folding and Binding. J. Am. Chem. Soc. 2021, 143, 14540–14550. 10.1021/jacs.1c04214. 34473923

[ref6] Krois A. S.; Park S.; Martinez-Yamout M. A.; Dyson H. J.; Wright P. E. Mapping Interactions of the Intrinsically Disordered C-Terminal Regions of Tetrameric p53 by Segmental Isotope Labeling and NMR. Biochemistry 2022, 61, 2709–2719. 10.1021/acs.biochem.2c00528. 36380579 PMC9788666

[ref7] Fuxreiter M. Electrostatics tunes protein interactions to context. Proc. Natl. Acad. Sci. U.S.A. 2022, 119, e2209201119 10.1073/pnas.2209201119. 35858387 PMC9351485

[ref8] Barrera-Vilarmau S.; Teixeira J. M. C.; Fuxreiter M. Protein interactions: anything new?. Essays Biochem. 2022, 66, 821–830. 10.1042/EBC20220044. 36416856 PMC9760424

[ref9] Karlsson E.; Schnatwinkel J.; Paissoni C.; Andersson E.; Herrmann C.; Camilloni C.; Jemth P. Disordered regions flanking the binding interface modulate affinity between CBP and NCOA. J. Mol. Biol. 2022, 434, 167643 10.1016/j.jmb.2022.167643. 35605677

[ref10] Kjaergaard M.; Teilum K.; Poulsen F. M. Conformational selection in the molten globule state of the nuclear coactivator binding domain of CBP. Proc. Natl. Acad. Sci. U.S.A. 2010, 107, 12535–12540. 10.1073/pnas.1001693107. 20616042 PMC2906600

[ref11] Kjaergaard M.; Poulsen F. M.; Teilum K. Is a malleable protein necessarily highly dynamic? The hydrophobic core of the nuclear coactivator binding domain is well ordered. Biophys. J. 2012, 102, 1627–1635. 10.1016/j.bpj.2012.02.014. 22500763 PMC3318130

[ref12] Kjaergaard M.; Andersen L.; Nielsen L. D.; Teilum K. A folded excited state of ligand-free nuclear coactivator binding domain (NCBD) underlies plasticity in ligand recognition. Biochemistry 2013, 52, 1686–1693. 10.1021/bi4001062. 23373423

[ref13] Dogan J.; Toto A.; Andersson E.; Gianni S.; Jemth P. Activation Barrier-Limited Folding and Conformational Sampling of a Dynamic Protein Domain. Biochemistry 2016, 55, 5289–5295. 10.1021/acs.biochem.6b00573. 27542287

[ref14] Demarest S. J.; Martinez-Yamout M.; Chung J.; Chen H.; Xu W.; Dyson H. J.; Evans R. M.; Wright P. E. Mutual synergistic folding in recruitment of CBP/p300 by p160 nuclear receptor coactivators. Nature 2002, 415, 549–553. 10.1038/415549a. 11823864

[ref15] Demarest S. J.; Deechongkit S.; Dyson H. J.; Evans R. M.; Wright P. E. Packing, specificity, and mutability at the binding interface between the p160 coactivator and CREB-binding protein. Protein Sci. 2004, 13, 203–210. 10.1110/ps.03366504. 14691235 PMC2286511

[ref16] Mirdita M.; Schütze K.; Moriwaki Y.; Heo L.; Ovchinnikov S.; Steinegger M. ColabFold: making protein folding accessible to all. Nat. Methods 2022, 19, 679–682. 10.1038/s41592-022-01488-1. 35637307 PMC9184281

[ref17] Jemth P.; Karlsson E.; Vögeli B.; Guzovsky B.; Andersson E.; Hultqvist G.; Dogan J.; Güntert P.; Riek R.; Chi C. N. Structure and dynamics conspire in the evolution of affinity between intrinsically disordered proteins. Sci. Adv. 2018, 4, eaau4130 10.1126/sciadv.aau4130. 30397651 PMC6200366

[ref18] Brown C. J.; Johnson A. K.; Daughdrill G. W. Comparing models of evolution for ordered and disordered proteins. Mol. Biol. Evol. 2010, 27, 609–621. 10.1093/molbev/msp277. 19923193 PMC2822292

[ref19] Hultqvist G.; Åberg E.; Camilloni C.; Sundell G. N.; Andersson E.; Dogan J.; Chi C. N.; Vendruscolo M.; Jemth P. Emergence and evolution of an interaction between intrinsically disordered proteins. Elife 2017, 6, e16059 10.7554/eLife.16059. 28398197 PMC5419745

[ref20] McLysaght A.; Hokamp K.; Wolfe K. H. Extensive genomic duplication during early chordate evolution. Nat. Genet. 2002, 31, 200–204. 10.1038/ng884. 12032567

[ref21] Smith J. J.; Kuraku S.; Holt C.; Sauka-Spengler T.; Jiang N.; Campbell M. S.; Yandell M. D.; Manousaki T.; Meyer A.; Bloom O. E.; Morgan J. R.; Buxbaum J. D.; Sachidanandam R.; Sims C.; Garruss A. S.; Cook M.; Krumlauf R.; Wiedemann L. M.; Sower S. A.; Decatur W. A.; Hall J. A.; Amemiya C. T.; Saha N. R.; Buckley K. M.; Rast J. P.; Das S.; Hirano M.; McCurley N.; Guo P.; Rohner N.; Tabin C. J.; Piccinelli P.; Elgar G.; Ruffier M.; Aken B. L.; Searle S. M. J.; Muffato M.; Pignatelli M.; Herrero J.; Jones M.; Brown C. T.; Chung-Davidson Y.-W.; Nanlohy K. G.; Libants S. V.; Yeh C.-Y.; McCauley D. W.; Langeland J. A.; Pancer Z.; Fritzsch B.; de Jong P. J.; Zhu B.; Fulton L. L.; Theising B.; Flicek P.; Bronner M. E.; Warren W. C.; Clifton S. W.; Wilson R. K.; Li W. Sequencing of the sea lamprey (*Petromyzon marinus*) genome provides insights into vertebrate evolution. Nat. Genet. 2013, 45, 415–421. 10.1038/ng.2568. 23435085 PMC3709584

[ref22] Karlsson E.; Lindberg A.; Andersson E.; Jemth P. High affinity between CREBBP/p300 and NCOA evolved in vertebrates. Protein Sci. 2020, 29, 1687–1691. 10.1002/pro.3868. 32329110 PMC7314397

[ref23] Bugge K.; Brakti I.; Fernandes C. B.; Dreier J. E.; Lundsgaard J. E.; Olsen J. G.; Skriver K.; Kragelund B. B. Interactions by Disorder - A Matter of Context. Front. Mol. Biosci. 2020, 7, 110 10.3389/fmolb.2020.00110. 32613009 PMC7308724

[ref24] Malagrinò F.; Pennacchietti V.; Santorelli D.; Pagano L.; Nardella C.; Diop A.; Toto A.; Gianni S. On the Effects of Disordered Tails, Supertertiary Structure and Quinary Interactions on the Folding and Function of Protein Domains. Biomolecules 2022, 12, 209 10.3390/biom12020209. 35204709 PMC8961636

[ref25] Ortega-Alarcon D.; Claveria-Gimeno R.; Vega S.; Jorge-Torres O. C.; Esteller M.; Abian O.; Velazquez-Campoy A. Stabilization Effect of Intrinsically Disordered Regions on Multidomain Proteins: The Case of the Methyl-CpG Protein 2, MeCP2. Biomolecules 2021, 11, 1216 10.3390/biom11081216. 34439881 PMC8391517

[ref26] Laursen L.; Karlsson E.; Gianni S.; Jemth P. Functional interplay between protein domains in a supramodular structure involving the postsynaptic density protein PSD-95. J. Biol. Chem. 2020, 295, 1992–2000. 10.1074/jbc.RA119.011050. 31831623 PMC7029118

[ref27] Brodsky S.; Jana T.; Barkai N. Order through disorder: The role of intrinsically disordered regions in transcription factor binding specificity. Curr. Opin. Struct. Biol. 2021, 71, 110–115. 10.1016/j.sbi.2021.06.011. 34303077

[ref28] González-Foutel N. S.; Glavina J.; Borcherds W. M.; Safranchik M.; Barrera-Vilarmau S.; Sagar A.; Estaña A.; Barozet A.; Garrone N. A.; Fernandez-Ballester G.; Blanes-Mira C.; Sánchez I. E.; de Prat-Gay G.; Cortés J.; Bernadó P.; Pappu R. V.; Holehouse A. S.; Daughdrill G. W.; Chemes L. B. Conformational buffering underlies functional selection in intrinsically disordered protein regions. Nat. Struct. Mol. Biol. 2022, 29, 781–790. 10.1038/s41594-022-00811-w. 35948766 PMC10262780

[ref29] Sievers F.; Wilm A.; Dineen D.; Gibson T. J.; Karplus K.; Li W.; Lopez R.; McWilliam H.; Remmert M.; Söding J.; Thompson J. D.; Higgins D. G. Fast, scalable generation of high-quality protein multiple sequence alignments using Clustal Omega. Mol. Syst. Biol. 2011, 7, 539 10.1038/msb.2011.75. 21988835 PMC3261699

[ref30] Edgar R. C. MUSCLE: multiple sequence alignment with high accuracy and high throughput. Nucleic Acids Res. 2004, 32, 1792–1797. 10.1093/nar/gkh340. 15034147 PMC390337

[ref31] Jumper J.; Evans R.; Pritzel A.; Green T.; Figurnov M.; Ronneberger O.; Tunyasuvunakool K.; Bates R.; Žídek A.; Potapenko A.; Bridgland A.; Meyer C.; Kohl S. A. A.; Ballard A. J.; Cowie A.; Romera-Paredes B.; Nikolov S.; Jain R.; Adler J.; Back T.; Petersen S.; Reiman D.; Clancy E.; Zielinski M.; Steinegger M.; Pacholska M.; Berghammer T.; Bodenstein S.; Silver D.; Vinyals O.; Senior A. W.; Kavukcuoglu K.; Kohli P.; Hassabis D. Highly accurate protein structure prediction with AlphaFold. Nature 2021, 596, 583–589. 10.1038/s41586-021-03819-2. 34265844 PMC8371605

[ref32] Dogan J.; Schmidt T.; Mu X.; Engström Å.; Jemth P. Fast association and slow transitions in the interaction between two intrinsically disordered protein domains. J. Biol. Chem. 2012, 287, 34316–34324. 10.1074/jbc.M112.399436. 22915588 PMC3464538

[ref33] Dogan J.; Mu X.; Engström Å.; Jemth P. The transition state structure for coupled binding and folding of disordered protein domains. Sci. Rep. 2013, 3, 2076 10.1038/srep02076. 23799450 PMC3691887

