# Supplementary material for: Intrinsically Disordered Flanking Regions Increase the Affinity of a Transcriptional Coactivator Interaction across Vertebrates
Source: Biochemistry. 2023 Aug 30;62(18):2710–6. doi: 10.1021/acs.biochem.3c00285 (PMC10515491; doi:10.1021/acs.biochem.3c00285)
Supplement: Supplementary file 1 — bi3c00285_si_001.pdf [file bi3c00285_si_001.pdf]

Supporting Information

Intrinsically disordered flanking regions increase affinity of a transcriptional co-activator interaction across vertebrates

Elin Karlsson[1,*], Carl Ottosson[1], Weihua Ye[1], Eva Andersson[1], and Per Jemth[1,*]

[1]Department of Medical Biochemistry and Microbiology, Uppsala University, BMC Box 582, SE-75123 Uppsala, Sweden

*Correspondence: elincbkarlsson@gmail.com

*Correspondence: per.jemth@imbim.uu.se

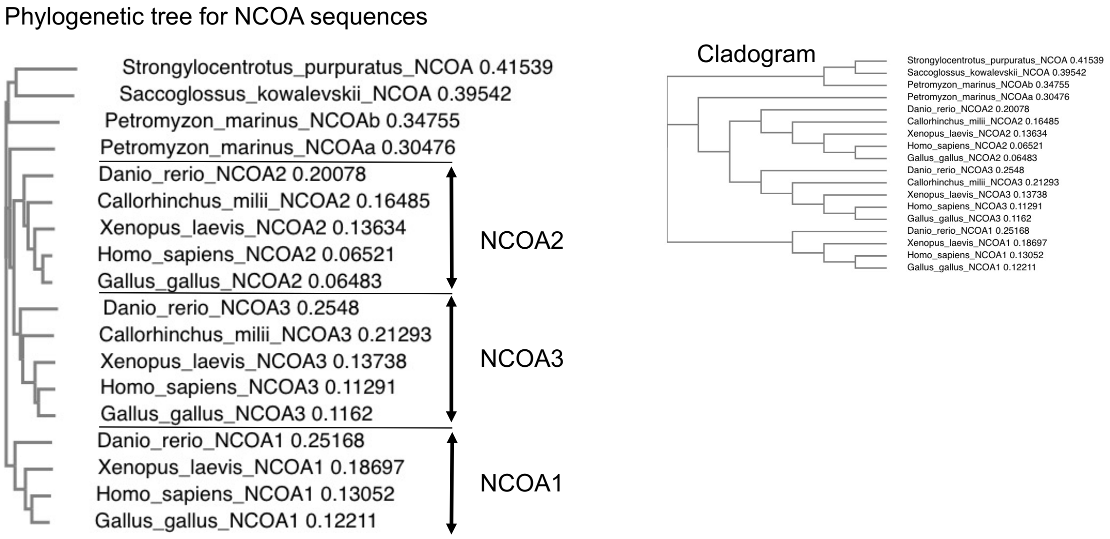

Figure S1. Phylogenetic tree for NCOA paralogs based on alignment of full-length protein sequences. NCOA1, NCOA2 and NCOA3 form well defined clades. NCOA2 and NCOA3 group together suggesting that they arose in the second genome duplication while NCOA1 diverged from the ancestral NCOA2/3 in the first genome duplication. NCOAb from *P. marinus* group with the non-vertebrate deuterostomes while *P. marinus* NCOAa is closer to the vertebrate NCOAs. The ambiguous positions of NCOAa and NCOAb likely reflects that they diverged from vertebrate NCOAs before the latter adapted to their present-day functions. The cladogram is shown to clearly depict the branching at the roots of the phylogenetic tree. The alignment and tree were done with Muscle.

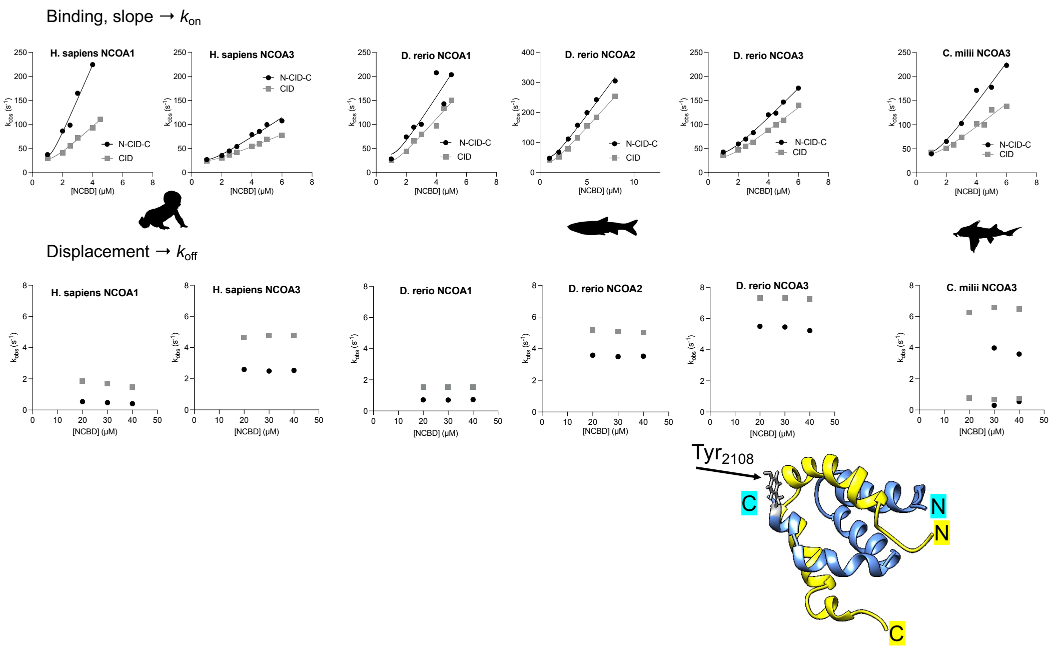

Figure S2. Observed rate constants from stopped flow experiments. Kinetic data for all NCBD/CID complexes included in the study. A second kinetic phase was detected in displacement experiments with *C. milii* NCOA3 NCBD and both N-CID-C and CID. We used the larger k_{obs} values as estimates for k_{off}. Tyr2108 in NCBD, indicated by an arrow, was mutated to Trp in each NCBD variant to obtain a fluorescent probe for the kinetic experiments.

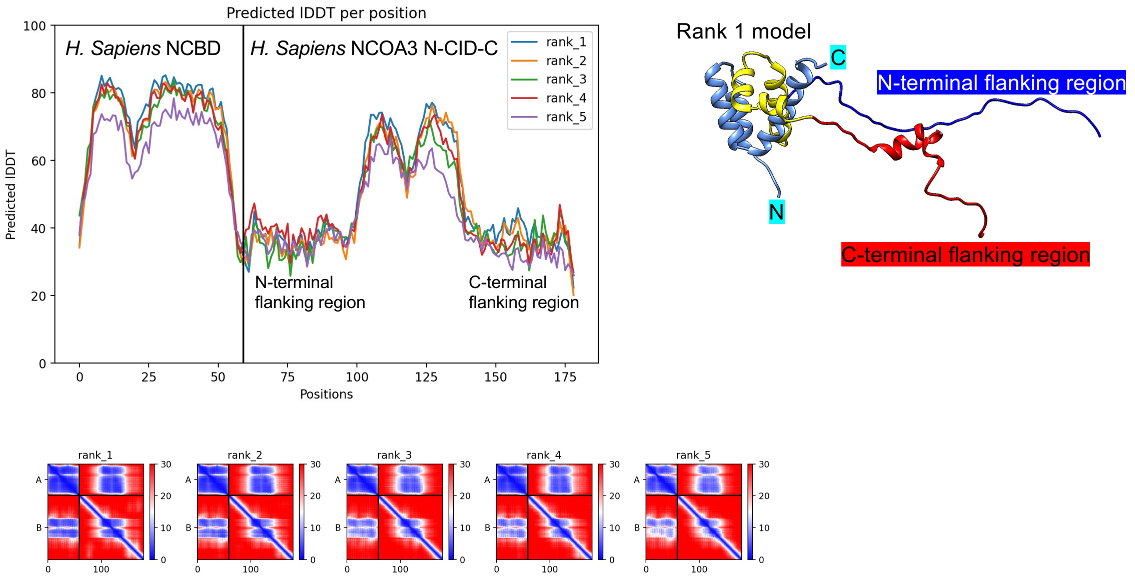

Figure S3. Confidence of the ColabFold prediction. The prediction was performed for the human complex between NCBD from CREB-binding protein and CID from NCOA3. The low predicted IDDT scores in the N- and C-terminal flanking regions of CID suggest that these regions are intrinsically disordered.
